# Diagnostic management of blastic plasmacytoid dendritic cell neoplasm (BPDCN) in close interaction with therapeutic considerations

**DOI:** 10.1007/s00277-023-05587-7

**Published:** 2024-01-09

**Authors:** Evgenii Shumilov, Paolo Mazzeo, Susanne Ghandili, Axel Künstner, Sören Weidemann, Yara Banz, Philipp Ströbel, Matthias Pollak, Lina Kolloch, Helmut Beltraminelli, Andrea Kerkhoff, Jan-Henrik Mikesch, Christoph Schliemann, Detlef Haase, Gerald Wulf, Myriam Legros, Georg Lenz, Laurence Feldmeyer, Thomas Pabst, Hanno Witte, Niklas Gebauer, Ulrike Bacher

**Affiliations:** 1 Department of Medicine A for Hematology, Oncology and Pneumology, University Hospital Muenster, Muenster, Germany; 2 Clinics of Hematology and Medical Oncology, INDIGHO Laboratory, University Medical Center Goettingen (UMG), Goettingen, Germany; 3 Department of Oncology, Hematology and Bone Marrow Transplantation With Section Pneumology, University Medical Center Hamburg-Eppendorf, Hamburg, Germany; 4 Medical Systems Biology Group, Luebeck Institute of Experimental Dermatology, University of Luebeck, Luebeck, Germany; 5 Institute of Pathology, University Medical Center Hamburg-Eppendorf, Hamburg, Germany; 6 Institute of Pathology, University of Bern, Bern, Switzerland; 7 Institute of Pathology, University Medical Center Goettingen, Goettingen, Germany; 8 Department of Hematology and Central Hematology Laboratory, Inselspital, Bern University Hospital, University of Bern, Bern, Switzerland; 9 Dermatopathology Department, Ente Ospedaliero Cantonale (EOC), Locarno, Switzerland; 10 Department of Hematology and Medical Oncology, University Medical Center Goettingen (UMG), Goettingen, Germany; 11 Department of Dermatology, Inselspital, Bern University Hospital, University of Bern, Bern, Switzerland; 12 Department of Medical Oncology, Bern University Hospital, University of Bern, InselspitalBern, Switzerland; 13 Department for Hematology and Oncology, University Hospital Schleswig-Holstein, Campus Luebeck, Luebeck, Germany; 14 Department for Hematology and Oncology, Bundeswehrkrankenhaus Ulm, Ulm, Germany

**Keywords:** Blastic plasmacytoid dendritic cell neoplasm (BPDCN), Comprehensive diagnostics, Flow cytometry, Immunohistochemistry, CD123, Tagraxofusp

## Abstract

Blastic plasmacytoid dendritic cell neoplasm (BPDCN), a rare malignancy derived from plasmacytoid dendritic cells, can mimic both acute leukemia and aggressive T-cell lymphoma. Therapy of this highly aggressive hematological disease should be initiated as soon as possible, especially in light of novel targeted therapies that have become available. However, differential diagnosis of BPDCN remains challenging. This retrospective study aimed to highlight the challenges to timely diagnoses of BPDCN. We documented the diagnostic and clinical features of 43 BPDCN patients diagnosed at five academic hospitals from 2001–2022. The frequency of BPDCN diagnosis compared to AML was 1:197 cases. The median interval from the first documented clinical manifestation to diagnosis of BPDCN was 3 months. Skin (65%) followed by bone marrow (51%) and blood (45%) involvement represented the most common sites. Immunophenotyping revealed CD4 + , CD45 + , CD56 + , CD123 + , HLA-DR + , and TCL-1 + as the most common surface markers. Overall, 86% (e.g. CD33) and 83% (e.g., CD7) showed co-expression of myeloid and T-cell markers, respectively. In the median, we detected five genomic alterations per case including mutational subtypes typically involved in AML: DNA methylation (70%), signal transduction (46%), splicing factors (38%), chromatin modification (32%), transcription factors (32%), and RAS pathway (30%), respectively. The contribution of patients (30%) proceeding to any form of upfront stem cell transplantation (SCT; autologous or allogeneic) was almost equal resulting in beneficial overall survival rates in those undergoing allogeneic SCT (*p* = 0.0001). BPDCN is a rare and challenging entity sharing various typical characteristics of other hematological diseases. Comprehensive diagnostics should be initiated timely to ensure appropriate treatment strategies.

## Introduction

Blastic plasmacytoid dendritic cell neoplasm (BPDCN) is a rare hematological malignancy that frequently shares key characteristics of acute leukemias and aggressive T-cell lymphoma. The term BPDCN was initially introduced within acute myeloid leukemia (AML)-related precursor neoplasms by the WHO in 2008 [1] and reclassified in 2016 as a separate entity within the spectrum of neoplasms[2]. BPDCN is derived from plasmacytoid dendritic cells (pDC) or earlier precursors (cDC) and typically presents with an aggressive clinical course [3, 4]. BPDCN occurs across all age groups with the peak among patients above 60 years of age, with around three-quarters of patients being male [5–7]. Skin lesions represent the most common clinical manifestations (70–100% of patients) and are typically accompanied by either simultaneous or subsequent involvement of bone marrow (BM; in roughly 60% of cases) and peripheral blood (PB; approximately 15–28% of cases) [6–9]. Along this line, tropism to lymph nodes (39%) and less often to visceral organs (21%) as well as the central nervous system (CNS) (4–9%) have also been documented [5, 7, 10–12].

Durable remissions can only be achieved in BPDCN patients with response to induction therapies followed by hematopoietic stem cell transplantation (SCT) as consolidation [13–15]. After allogeneic SCT, 3-year overall survival (OS) rates of 59–71% have been reported [7, 16]. Although data on the efficacy of autologous SCT are inconsistent for BPDCN patients [7, 11, 16], it is considered an alternative strategy when allogeneic SCT is not feasible. Recently, novel targeted therapies such as the BCL2-inhibitor venetoclax, the hypomethylating agent 5-azacytidine, and the anti-CD123-antibody-toxin-conjugate tagraxofusp have shown promising results in therapy-naive and/or relapsed/refractory BPDCN [17, 18].

Since BPDCN shares various common clinical and diagnostic features with acute leukemias and aggressive T-cell lymphomas, differential diagnosing is challenging and requires a thorough diagnostic workup as well as awareness by the treating physician. Particularly, the detection of the defining immunophenotype is essential. Along this line, immunohistochemistry and multicolor multiparameter flow cytometry (FC) play a crucial role in diagnosing BPDCN, especially regarding the evaluation of BM samples. Potentially, it enables the discrimination of BPDCN from acute myeloid leukemia (AML), NK/T-cell lymphoma, and/or other T-cell lymphomas. Acknowledging the importance of immunophenotypic characterization in BPDCN, additional challenges arise when neoplastic cells do not meet the typical CD4 + /CD56 + /CD123 + /HLA-DR + /TCL1/CD303 + profile [8, 19]. Furthermore, BPDCN cells frequently share markers from other lineages (e.g., CD33, TdT, CD79a, CD2, and CD7) [20, 21] and show atypical immunophenotypic profiles (e.g., negativity for CD56 or CD4) [22]. Furthermore, BPDCN-like phenotype appears to confer a negative prognosis for distinct myeloid malignancies [23]. Recently, Khanlari et al. demonstrated clonal hematopoiesis (CH) can present in nearly two-thirds of BPDCN patients. Yet, about one-third of these patients with BM CH had myelodysplastic syndrome (MDS), chronic myelomonocytic leukemia (CMML), or myeloproliferative neoplasm (MPN), diagnosed either prior to BPDCN or at the same time when the patient underwent BM staging for BPDCN involvement [24]. Thus, BPDCN can closely resemble several hematological neoplasms—both clinically as well as diagnostically—and discrimination of cases with features of BPDCN from definitive BPDCN can be very tricky [25].

Molecular and cytogenetic analyses have extended the diagnostic spectrum of BPDCN. Most BPDCN cases have chromosomal abnormalities detectable by conventional karyotyping with around one-third of patients presenting with three or more aberrations [26]. Following the introduction of next-generation sequencing (NGS) techniques, multiple mutations have been recurrently identified in BPDCN patients including known oncogenes (e.g., *NRAS*, *KRAS*), tumor repressor genes (e.g., *TP53*), epigenetic regulators (e.g., *IDH1*/-*2*, *TET2*), and splicing factor genes (e.g., *ZRSR2*, *SRSF2*, and *SF3B1*) [27–29]. The presence of multiple mutations and/or mutations in the DNA methylation pathway at first diagnosis may be associated with poor patient outcomes [30].

Traditionally, histopathology and immunohistochemistry (IHC) are the cornerstones for diagnosing BPDCN. However, this complex disorder remains underdiagnosed and the frequency of BPDCN is underestimated. In light of the novel targeted treatment option—tagraxofusp, an anti-CD123 conjugated cytotoxic agent approved in November 2021 by EMA [31], as well as anti-CD123 CAR T-cells tested in preclinical models and early clinical trials [32, 33], the importance of a correct and timely diagnosis is essential. Tagraxofusp is a fusion protein consisting of interleukin 3 (IL-3) fused to diphtheria toxin. It eliminates pDC by binding to their IL-3 receptors (CD123), gaining entrance to the cells, and then blocking protein synthesis due diphtheria toxin portion inhibiting eukaryotic elongation factor 2 [34].

This retrospective study aimed to summarize current challenges and options for accurate differential diagnosis of BPDCN analyzing all patients diagnosed with BPDCN in five German and Swiss university cancer centers in the last two decades.

## Patients and methods

This multicenter retrospective study enrolled 43 consecutive patients with BPDCN diagnosed and/or treated between 2001 and 2022 in five academic hospitals: University Hospital/Inselspital Bern, Switzerland, as well as University Hospital Göttingen, University Hospital Münster, University Hospital Hamburg, and University Hospital of Schleswig–Holstein, Campus Lübeck, Germany. Identification of the cases mentioned above was verified by IHC and/or FC leading to the diagnosis of BPDCN. Clinical data was gathered from the medical records electronic patient files, electronic databases of the hospitals, or supplemented by additional patient-related documents. The University Hospital of Schleswig–Holstein, Campus Lübeck, included only patients from the reference center for lymph node pathology and hematopathology in Lübeck. The retrospective data analysis was approved by decisions of the local ethics committees (Ethics Committee of the Westphalia-Lippe Medical Association no. 2023–071-f-S; Ethics Committee of the University Medical Center Göttingen no. 17/4/23 Ü; Ethics Committee of the Hamburg Medical Association no. 2022–300263-BO-bet; and Ethics Committee of the University Lübeck no. 18–311).

### Flow cytometry

BM aspirate specimens (in heparin) and peripheral blood specimens (in EDTA) were processed within 24 h of collection using a standard lyse/wash technique (BDLyse™, BD Biosciences, San Diego, CA, USA). The samples were recorded on a FACSCanto II (Münster; Bern), FACS Lyric instrument (8-color, BD Biosciences) (Bern), and Navios EX flow cytometer (Hamburg). At the time of initial diagnosis, the comprehensive flow cytometry panel included lineage-defining markers for B, T, myeloid, and monocytic cells, as well as markers (CD4, CD123, HLA-DR, CD56) necessary for the diagnosis of BPDCN with only subtle differences between the four centers. The flow cytometry results from the University Hospital of Schleswig–Holstein, Campus Lübeck, were not available and therefore not included in the analysis.

### Histopathology and immunohistochemistry

BM biopsy specimens were routinely fixed in either 4 or 10% neutral buffered formalin solution, decalcified overnight using EDTA (DecalMATE, Leica Biosystems, Muttenz, Switzerland), and embedded in paraffin (all centers). Two-micrometer sections of formalin-fixed and paraffin-embedded (FFPE) blocks were stained by hematoxylin and eosin (H&E), Giemsa, silver staining, periodic acid Schiff (PAS), and Turnbull’s blue iron stain. Standard IHC stains were performed using the fully automated stainer BOND-III systems (Leica Biosystems) and consisted of a standard panel of CD71, CD42b, myeloperoxidase (MPO), CD34, CD117, CD3, CD20, and CD138 as well as additional stains as needed for diagnosis including CD4, CD5, CD7, CD8, CD11b, CD14, CD15, CD33, CD45, CD56, CD68, CD79a, CD123, lysozyme, TdT, and HLA-DR.

### Dermatopathology

Skin biopsy specimens were routinely fixed in a 10% neutral-buffered formalin solution, and embedded in paraffin. The 4.5-µm sections of FFPE blocks were stained by H&E. Immunohistochemical analysis using the biotin-free alkaline phosphatase method (Leica Biosystems, Newcastle, UK) was performed using the fully automated stainer BOND-III systems (Leica Biosystems) with the following primary antibodies: CD3 (clone LN10, Leica Biosystems), CD4 (clone 4B12, DakoCytomation, Glostrup, Denmark), CD5 (clone 4C7, Leica Biosystems), CD20 (clone LR26, Leica Biosystems), CD56 (clone NCAM, Leica Biosystems), CD68 (clone 514H12, Leica Biosystems), CD123 (clone BR4MS, Novocastra, Leica Biosystems), TCL-1 (clone MRQ-7, Cell Marque, CA Rocklin, USA), TIA-1 (Biocare Medical, CA Pacheo, USA), and MPO (Cell Marque). Irrelevant IgG subclass‒matched antibodies were used for negative controls.

### Cytogenetics and molecular genetics

Details of cultivation, chromosomal banding on G-banded chromosomes procedure, and processing have been described elsewhere [35–38]. Karyotypes were documented according to ISCN [39, 40]. Sequencing analysis including NGS was performed using targeted NGS panels with the most recurrent mutated genes in myeloid neoplasms (Bern—sequencing on the Ion Torrent S5 platform with the Oncomine Myeloid Research Panel comprising 40 genes and hotspots; Göttingen and Hamburg—sequencing on Illumina with targeted DNA panels including up to 54 genes [41]; Münster—sequencing on NovaSeq with 13 genes [27]). The cut-off of variant allele frequency (VAF) for the interpretation of variants was ≥ 5%. Lower allele frequencies were considered for hotspot regions in genes with clear clinical relevance, including *TP53*. The University Hospital of Schleswig–Holstein, Campus Lübeck, applied whole-exome sequencing (WES) and RNA-Sequencing (RNA-Seq) following library preparation using Agilent SureSelect Human All Exon V6 library preparation kit (Agilent Technologies) and NEBNext® UltraT Directional RNA Library Prep Kit (New England BioLabs), respectively. Sequencing was performed on a NovaSeq platform (Illumina) at Novogene (UK) Co. as described previously [42]. Tumor whole exome libraries were sequenced to a median depth of 119 × (mean 129 ± 54 SD), and normal libraries reached a median depth of 67 × (mean 83 ± 36 SD).

## Results

### Clinical characteristics of BPDCN patients

Clinical and laboratory findings of the 43 patients diagnosed with BPDCN are presented in Table 1. The median age at diagnosis was 70 years (range, 15–91) with a male predominance (33/43; 77%). The median interval from the first documented clinical manifestation to the diagnosis of BPDCN was 3 months (range, 1–6 months) in patients with available data (14/43). The clinical presentations of BPDCN were as follows: skin lesions (28/43; 65%), BM involvement (22/46; 51%), PB (9 out of 20 cases with available data; 45%), lymphadenopathy (16/43; 31%), and hepato- and/or splenomegaly (7/43; 16%). Notably, three patients (7%) had also CNS manifestations. Nine out of 43 patients (21%) had a history of second hematologic malignancy documented either preceding BPDCN (*n* = 5/9) or in coincidence (4/9)—MDS (*n* = 4), CMML (*n* = 2), or various T-cell lymphomas (*n* = 4). Two of these patients initially suffered from T-cell lymphoma followed by MDS and CMML, respectively.

**Table 1:** Clinical characteristics of the 43 BPDCN patients at diagnosis in this analysis

Parameter	All patients (*n* = 43)	
Gender (male), *n* (%)	33/43	77%
Median age, years (range)	70	15–91
**Disease classification**		
**BPDCN, *n*** **(%)**	***n* = 43**	**100%**
**Manifestation at diagnosis**		
Skin	28	65%
Bone marrow	22	51%
Peripheral blood	9/20	45%
Lymphadenopathy	16	31%
Hepatosplenomegaly	7	16%
CNS	3	7%
Others	5	12%
**Second hematologic malignancy**	8	19%
Synchrone with BPDCN	4	9%
Preceding BDPCN	5	12%
**Type of second hematologic malignancy**	9	21%
MDS/CMML*	7	16%
Hodgkin lymphoma	2	5%
T-cell lymphoma*	2	5%
**Cytogenetics at diagnosis**	***n* = 11**	**100%**
Normal karyotype	5	45%
Distinct aberrations	4	37%
Complex karyotype (≥ 3 aberrations)	2	18%
**Copy number variation by array-based procedure**	***n* = 26**	**100%**
Detected	15	58%
Chromosome 7 involvement	12	46%
Chromosome 9 involvement	9	35%
Chromosome 5 involvement	5	19%
Chromosome 10 involvement	2	8%
**Gene fusions number by RNA-seq**	***n* = 26**	**100%**
Median gene fusion number, *n* (range)	5	0–54
**Molecular genetics at diagnosis**	***n* = 36**	**100%**
NGS	35	97%
PCR	1	3%
**Median number of mutated genes, *N* (range)**	5	0–13
**Involved gene groups**		
DNA methylation	26	72%
Signal transduction	17	47%
RNA splicing	14	39%
Chromatin modification	12	323%
Transcription	12	33%
RAS pathway	11	31%
*NPM1*	6	17%
Others	22	61%

^*^Two patients simultaneously with MDS or CMML and T-cell lymphoma

*M* male, *F* female, *BPDCN* blastic plasmacytoid dendritic cell neoplasm, *CNS* central nervous system, *MDS* myelodysplastic syndrome, *CMML* chronic myelomonocytic leukemia, *NGS* next generation sequencing, *PCR* polymerase chain reaction

### Pathology and Immunohistochemistry

The results of IHC are presented in Table 2. IHC was available in 42 of 43 BPDCN patients (98%) and performed either on the skin (22/42; 52%), bone marrow (12/42; 29%), lymph node (7/42; 17%), and adipose tissue (1/42; 2%) samples. If tested, more than 80% of all BPDCN cases expressed the following marker combination: CD4, CD56, CD123, CD303, and TLC-1. Yet, only a few patients were CD4 and CD123 negative (10% and 3%, respectively). The co-incidence of myeloid markers varied broadly with CD33 (86%) and CD117 (30%) being the most common ones. Co-expression of lymphoid markers such as CD3, TdT, and CD79a was found in 26%, 56%, and 70% of available cases.

**Table 2:** Results of immunohistochemistry (IHC) and flow cytometry of BPDCN patients in this studyImmunohistochemistry (IHC)

**Immunohistochemistry (IHC)**	**All patients (*n* = 42)**	
**IHC sample**	**No. of sample**	**%**
Skin biopsy	22	52%
Bone marrow	12	29%
Lymph node	7	17%
Adipose tissue	1	2%
**Immunohistochemical characteristics**		
**IHC marker**	**No. of pts**	**Positivity% (*n*, cases)**
CD123	39	97% (38/39)
CD4	40	90% (36/40)
CD56	41	100% (41/41)
CD45	6	100% (6/6)*
TCL1	31	81% (25/31)
CD303	3	100% (3/3)
CD33	29	86% (25/29)
CD34	30	17% (5/30)
CD68	5	20% (1/5)
CD117	30	30% (9/30)
MPO	34	6% (2/34)*
TdT	32	56% (18/32)
CD3	35	26% (9/35)*
CD19	4	0% (0/4)
CD20	10	10% (1/10)*
CD38	3	33% (1/3)*
CD79a	33	70% (23/33)
**Flow cytometry**	**All patients (*n* = 14)**	
**Sample materials**	**No. of sample**	**%**
Peripheral blood	4	29%
Bone marrow	10	71%
**Flow results**	**No. of pts**	**Positivity% (*n*, cases)**
CD123	11	100% (11/11)
CD4	11	100% (11/11)
CD56	11	100% (11/11)
CD45	8	87% (7/8)
TCL1	Not tested	
CD303	Not tested	
HLA-DR	9	100% (9/9)
CD11b	3	33% (1/3)*
CD13	5	40% (2/5)
CD14	4	25% (1/4)
CD15	4	25% (1/4)
CD33	8	63% (5/8)
CD34	8	25% (2/8)
CD117	7	57% (4/7)
MPO	7	29% (2/7)
TdT	2	0% (0/2)
CD2	7	43% (3/7)
CD3	8	0% (0/8)
CD7	6	83% (5/6)
CD19	5	0% (0/5)
CD20	2	0% (0/2)
CD38	3	100% (3/3)

^*^Weak expression only

*CD* cluster of differentiation, *MPO* myeloperoxidase, *TdT* terminal deoxynucleotidyl transferase, *TCL* T-cell leukemia/lymphoma 1, *HLA-DR* human leukocyte antigen-DR isotype

### Flow cytometry

FC findings from BM and/or PB are presented in Table 2. Samples of all BPDCN patients analyzed expressed CD4, CD56, CD123, and HLA-DR, in all tested cases. In accordance with IHC, myeloid markers such as CD33 and CD117 were most frequently present, 63% and 57%, respectively. Of note, 43% and 83% of cases demonstrated co-expression of T-cell markers such as CD2 and/or CD7, accordingly.

### Genetic characteristics

**Fig. 1:**
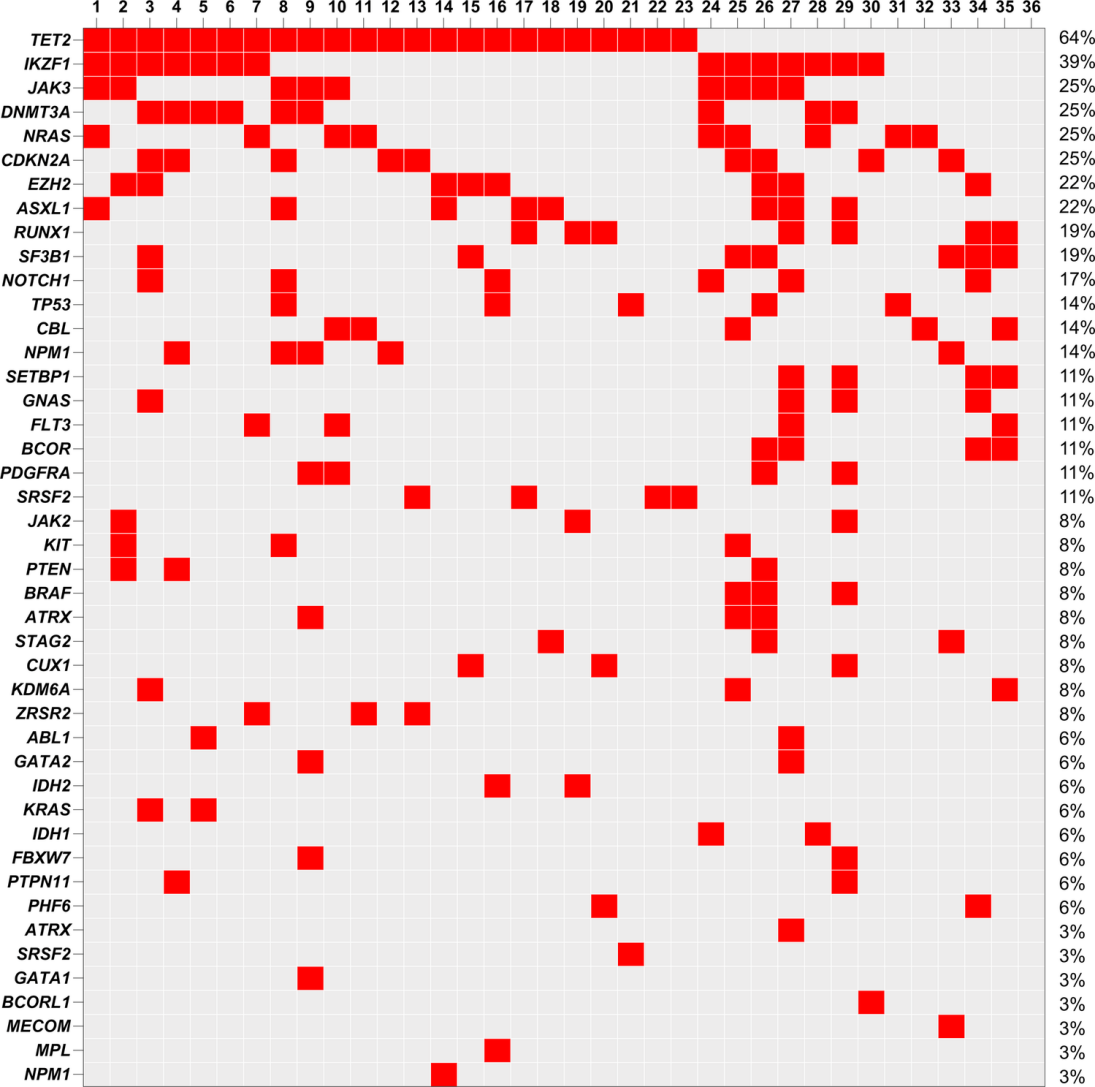
The heatmap visualizes the genetic profile across our patient cohort emphasizing the most recurrently mutated genes (in descending order) observed within our study

Conventional cytogenetic data for BM samples were available in 11 of 43 BPDCN cases (26%) (Table 1). Karyotyping revealed a normal karyotype in five patients (45%), whereas two patients (18%) showed a complex karyotype; the remaining patients (37%) presented either unbalanced cytogenetic aberrations including del(5q), del(7q), del(12p), and − Y or ins(8;6). Copy number variations (CNV) assessed by array-based procedure (OncoScan Array) were available for 26 patients, with 58% (15/26) of them showing cytogenetic changes predominantly in chromosome 7 (46%) and followed by aberrations in chromosome 9 (35%), 5 (19%), and 10 (8%) (Table 1). The median gene-fusion number in these 26 patients was 5. Data on molecular genetics were available in 37 out of 43 patients (35/37 NGS, 2/37 PCR). Sanger sequencing was performed on the two patients studied by PCR, covering a panel of 16 genes. The median number of molecular mutations in our cohort of patients was five, ranging from 0 to 13. The detected mutations were allocated to the following subgroups: DNA methylation (26/37; 70%), signal transduction (17/37; 46%), splicing factors (14/37 pts; 38%), chromatin modification (12/37; 32%), transcription factors (12/37; 32%), RAS pathway (11/37; 30%), *NPM1* (6/37; 16%), and others (22/37; 59%). The most frequent mutated genes are presented in Fig. 1.

### Clinical course and treatment outcomes

#### BPDCN patients

**Fig. 2:**
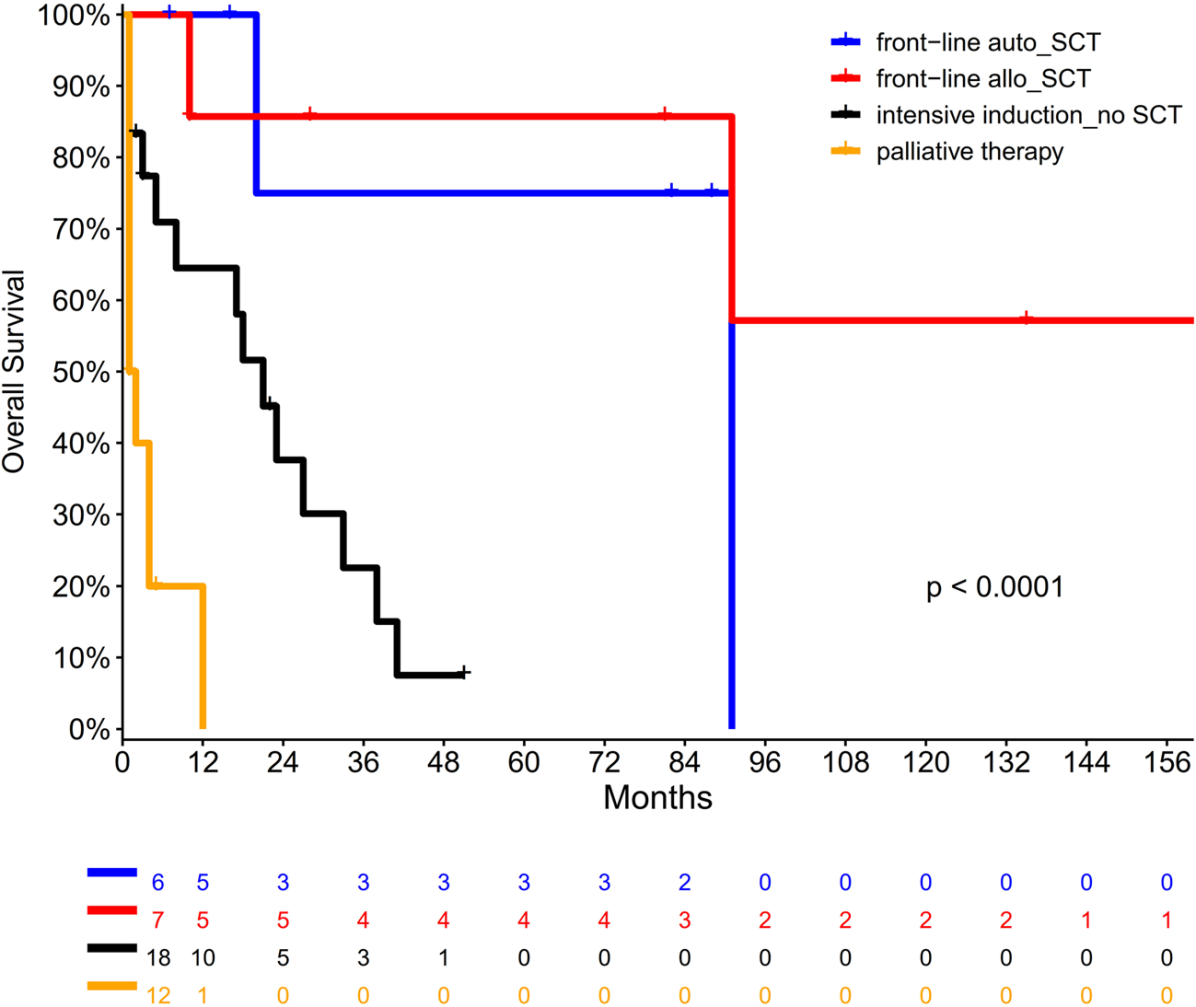
Overall survival in BPDCN patients depending on applied front-line treatment. BPDCN blastic plasmacytoid dendritic cell neoplasm, auto-SCT autologous stem cell transplantation, allo-SCT allogeneic stem cell transplantation, SCT stem cell transplantation

Treatment modalities applied in BPDCN patients are presented in Table 3. In detail, 18 patients (42%) were treated with leukemia- and 15 patients (35%) with lymphoma-based regimens, whereas three patients (7%) received CD123 targeting therapy with tagraxofusp. Two patients had local radiotherapy only due to isolated skin manifestations (5%). Four patients (9%) received best-supportive care (BSC) only after BPDCN diagnosis. Subsequently, 13 patients (30%), one of them without any induction treatment, underwent SCT, either allogeneic (7/13) or autologous SCT (6/13) in a front-line setting. Ten and three patients received subsequent second- and third-line therapy with 3 and 1 of them proceeding to salvage allogeneic SCT, respectively (for details see Table 3). With a median follow-up of 10 months (range, 1–229), 15 patients were still alive (35%), of the two patients who received radiotherapy only. The remaining patients with available data (*n* = 25) died either from relapsed/refractory (r/r) disease (16/25; 64%), infections (7/25; 28%), or other reasons (2/25; 8%). Accordingly, remission status at the last follow-up was as follows (available for 38 pts): complete remission (CR) (13/38; 34%), partial remission (PR) (2/38; 5%), and r/r disease (23/38; 61%). All considered patients received any cancer therapy at initial diagnosis (*n* = 39), those undergoing (autologous or allogeneic) SCT within a front-line treatment demonstrated significantly better overall survival in comparison to remaining patients (*p* = 0.0001) (Fig. 2).

**Table 3:** Therapy regimens and clinical outcomes among 43 BPDCN patients in this study

Parameter	All patients (*n* = 43)	
**Induction therapy**	*N*	%
**Acute leukemia-based regimen**	18	42%
Anthracycline/ARAC	5	12%
GMALL protocol	9	21%
5-Azacitidine	4	9%
**Lymphoma-based regimen**	15	35%
CHO(E)P	12	28%
DeVIC followed by RTX	2	5%
Platin-based induction	1	2%
**Tagraxofusp (anti-CD123 treatment)**	3	7%
**Local radiotherapy**	2	5%
**No induction therapy/direct allo-SCT1**	1	2%
**BSC**	4	9%
**Front-line stem cell transplantation (SCT1)**	13	30%
Allo-SCT1	7	16%
Auto-SCT1	6	14%
**Second-line therapy**	11	26%
Lymphoma-based regimen	3	7%
Acute leukemia-based regimen	5	12%
Local radiotherapy	2	5%
**Tagraxofusp**	1	2%
**Second-line stem cell transplantation (SCT2)**		
allo-SCT2	3	7%
**Third-line therapy**	3	7%
Lymphoma-based regimen	1	2%
Acute leukemia-based regimen	2	5%
**Third-line stem cell transplantation (SCT3)**		
allo-SCT3	1	2%
**Median follow-up after BDPCN diagnosis, months (range)**	10	(1–229)
**Survival status at last follow-up**		
Alive	15	35%
Dead	28	65%
**Causes of death (*n* = 25 available)**		
R/R disease	16	64%
Infection	7	28%
Others	2	8%
**Remission status at last follow-up (***n*** = 38 available)**		
CR	13	34%
PR	2	5%
PD	23	61%

*ARAC* cytarabine, *GMALL* German Multicenter Study Group for Adult Acute Lymphoblastic Leukemia, *CHO(E)P* cyclophosphamide hydroxydaunorubicin vincristine (etoposide) prednisone, *DeVIC* dexamethasone etoposide ifosfamide carboplatin, *RTX* rituximab, *allo-SCT1* first allogeneic stem cell transplantation, *BSC* best supportive care, *auto-SCT1* first autologous stem cell transplantation, *R/R* relapsed/refractory, *CR* complete hematologic remission, *PR* partial remission, *SD* stable disease

#### Illustration of comprehensive BPDCN diagnostics

**Fig. 3:**
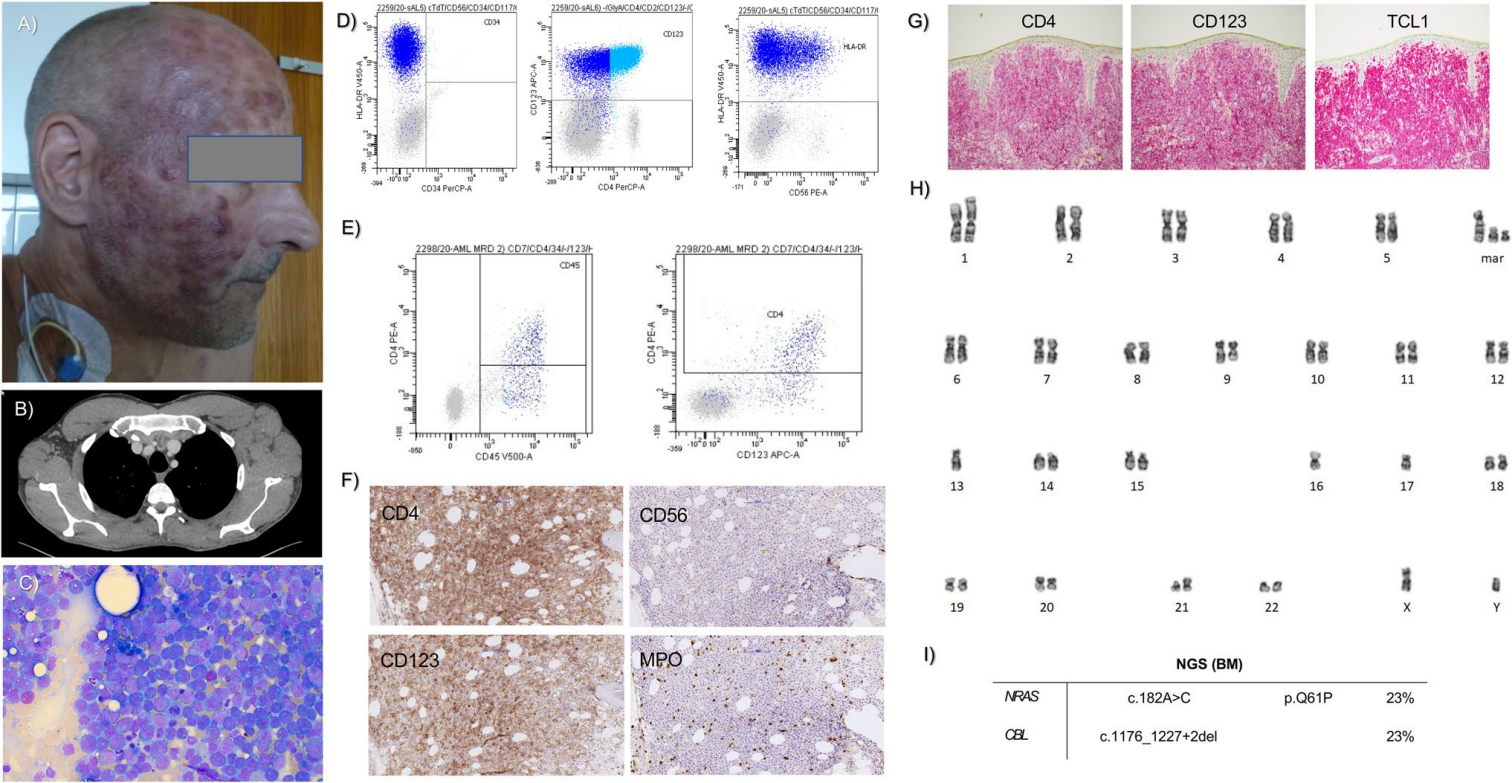
Comprehensive diagnostics of BPDCN. **A** Male patient with multiple skin lesions. **B** Axillary lymphadenopathy on CT. **C** BM aspirates with excess blast cells. **D** Flow cytometry (BM): CD4 + /CD56 + /CD123 + /HLA-DR + . **E** Flow cytometry (skin); CD4 + /CD45 + /CD123 + . **F** IHC (BM); CD4 + , CD56 + (weak), CD123 + , MPO − . **G** IHC (skin); CD4 + , CD123 + , TCL + . **H** Karyotype (BM); 46,XY,add(1) (p?36),add(9)(q?21), − 13,add(13)(p10), − 16, 17,add(21)(p10), + der(?)t(?;1)(?;q21), + 2mar[17] /46,XY[2]. **I** NGS (BM), % VAF; mutated *NRAS* and *CBL.* BM bone marrow, IHC immunohistochemistry, NGS next-generation sequencing, VAF variant allele frequency

An illustrative case presented in Fig. 3 exemplary demonstrates comprehensive diagnostics of BPDCN including skin and BM pathology, flow cytometry of PB and skin, cytogenetics, and molecular genetics from the BM of one of the patients.

#### Frequency of BPDCN in relation to acute myeloid leukemia in participating centers

Since 2009, we identified 17 BPDCN cases in contrast to 3157 AML patients documented as follows: University Hospital Hamburg 7:712, University Hospital Bern 4:872, and University Hospitals Göttingen and Münster 3:418 and 3:1055, respectively. The fifth center, University Hospital Lübeck, included patients from the reference center for lymph node pathology and hematopathology Lübeck and therefore was not considered for the analysis of BPDCN frequency. On average, one BPDCN case was diagnosed for every 197 AML cases.

## Discussion

With the introduction of tagraxofusp, interest in BPDCN has been increasing in the hemato-oncologic community in recent years. Focusing on the differential diagnostic difficulties that may arise in the context of BPDCN and the discrimination from other hematologic entities, we retrospectively evaluated BPDCN cases from five University cancer centers in Germany and Switzerland between the years 2001 and 2022 including different diagnostic methods.

The frequency of BDPCN is extremely rare and it can remain undiagnosed even in the era of modern comprehensive diagnostics. The clinical course is very heterogeneous ranging from skin involvement via lymphadenopathy to circulating blasts and cytopenias in the peripheral blood. As BPDCN cells are not homogeneously differentiated and do not emerge from a uniform pool of progenitors, they may co-express lymphoid and myeloid markers mimicking respective hematologic malignancies [43, 44]. Moreover, BPDCN frequently presents with several mutations at diagnosis similar to myeloid malignancies.

Remarkably, the ratio of a diagnosis of BPDCN compared to the diagnosis of AML was almost 1:200 cases in our centers. This highlights the rarity of BPDCN diagnosis even in large hemato-oncologic centers. In congruence with its rarity, our analysis revealed a median diagnostic delay of 3 months from the initial clinical presentation to confirmation of BPDCN diagnosis. The latter is critical since patients are diagnosed mostly with high tumor burden and worsening performance status potentially limiting subsequent therapy options.

The diagnostic algorithm in BPDCN should be based on thorough clinical history and examination, laboratory workup, and histopathology/immunophenotyping. Especially, immunophenotyping of the skin, peripheral blood, BM, or other manifestations can be essential in timely diagnosing BPDCN. The former consists of IHC and FC and suggests close cooperation of different medical disciplines, e.g., hemato-oncology, dermatology, histopathology, and dermatopathology. Our cases exhibited the characteristic BPDCN immunophenotype with positivity for CD4, CD45, CD56, CD123, HLA-DR, and TCL-1. Nevertheless, some cases were negative for CD4 and TCL-1. This suggests that the diagnosis of BPDCN represents a multimodal process in which the clinical presentation should be consistent with the majority of IHC/FC markers with some crucial markers sometimes being negative. As previously reported, this is conclusive with distinct mechanisms in the pathogenesis of BPDCN depending on the cell of origin hypothesis [42, 45].

Both myeloid and lymphoid markers were detectable by immunohistochemistry and/or flow cytometry to varying extents in our multicenter cohort. CD33 and CD117 were the most common antigens of myeloid origin, detectable in 86% and 57%, respectively. CD7, CD79a, TdT, and CD2 were the most frequently detected lymphoid markers in 83%, 70%, 56%, and 43% of cases. The possible explanation for that could be that dendritic cells can originate from myeloid and lymphoid progenitors [46, 47]. Following that, an expression of myeloid and/or B-/T-lymphoid markers can occur in BPDCN and must not exclude its diagnosis [26]. Thus, the above panel of classical BPDCN as well as pan-myeloid and lymphoid markers should be included in a comprehensive diagnostic workup when BPDCN is considered. It should be mentioned that CD4, CD45, CD56, CD123, HLA-DR, and TCL-1 panels are characteristic but still not entirely specific for BPDCN. Indeed, CD123 can be positive in myeloid blasts as well, although with weaker expression compared to BPDCN. TCL1 is also expressed in AML as well as in mature and immature B-cell malignancies and T-cell prolymphocytic leukemia [48]. Moreover, the combination of CD4, CD56, and CD123 can occur in AML and myeloid sarcoma with monocytic differentiation [49, 50]. Aiming to improve the sensitivity of immunophenotyping in the diagnostic of BPDCN, several additional markers have been proposed, e.g., TCF4 and CD304.[45, 49] Particularly, TCF4 is commonly expressed in BPDCN and is negative in other myeloid neoplasms including AML, while CD304 expression was found in all BPDCN cases investigated [45]. These markers should be tested as well if possible. Ideally, IHC should be always supported by FC and vice versa in BPDCN diagnostics. In our cohort and in line with previous reports, skin manifestation followed by BM and peripheral blood involvement were the most common clinical features of BPDCN [6–9]. Similar to acute leukemia, up to one-third of all BPDCN patients had lymphadenopathy and almost one-fifth had hepatosplenomegaly. Notably, 7% of patients additionally had CNS manifestations. In fact, the incidence of the latter among BPDCN may be totally underestimated as far as lumbar puncture has not historically been part of a diagnostic work-up in this entity. Particularly, several reports documented an occult CNS involvement ranging from 10 to 69% at the first diagnosis and/or relapse [51, 52]. Following that, we recommend screening lumbar punctures at diagnosis and relapse or in case of the new neurologic symptoms with subsequent immunophenotype testing of cerebro-spinal fluid (CSF). Prospectively, consistent CSF screening will lead to the optimal therapeutic strategy effectively addressing CNS manifestations in BPDCN, ensuring that CNS-directed therapy, e.g., intrathecal therapy or systemic methotrexat-containing regimens, will be administered in respective cases.[27, 53, 54].

Within our cohort, classical myeloid mutations affecting predominantly DNA methylation followed by signal transduction, splicing factors, chromatin modification, transcription factors, RAS-Pathway, and *NPM1* were frequently detected. These findings are in line with previous reports on molecular genetic characteristics of BPDCN [45, 55, 56]. Meanwhile, multiple pathways have been described on the molecular level in BPDCN. Preceding our current analysis, Künstner et al. characterized 47 BPDCN patients for mutational drivers, cytogenetic aberrations, and gene-expression profiles via WES and RNA-Seq with genome-wide copy-number analysis. Notably, multiple genes affected in BPDCN were shared with CMML, emphasizing a close relationship between these entities and surprisingly to a lesser extent with AML. Recently, the ontological assessment of RNA-Seq data revealed two BPDCN subtypes, a typical pDC-derived subtype (C1) and a (common) cDC-enriched subtype (C2), which were then shown to exhibit distinct mutational and clinical features [42]. Subsequently, Witte et al. [57] revealed a subset of significantly differentially methylated genes between these two clusters. Notably, pDC proliferations can occur in the context of AML and CMML as well. While these are often TCL1 positive, they usually lack mandatory BPDCN markers such as CD56. BPDCN-like cases with mutated *RUNX1* should be considered very carefully as far as *RUNX1* mutations are the most common somatic alterations in pDC-AML (> 70%) and much more common than in AML without pDC expansion and BPDCN [58, 59].

Several recent studies revealed a significant genomic heterogeneity between different BPDCN manifestations at a given time point [60–62]. These studies have highlighted the path of clonal evolution from a founding pDC clone originating from clonal hematopoiesis of indeterminate potential (CHiP) in the bone marrow, that is subsequently transformed in the skin by UV-radiation and subsequent loss of CDKN2A and/or activating mutations affecting the RTK/RAS pathway. From this stage, systemic dissemination occurs quickly and clonal evolution, shaped by therapeutic selection pressure generates even further mutational heterogeneity between different BPDCN manifestations. Of note, for the present study, we merely conducted DNA sequencing at one site per patient. In order to fully grasp clonal diversity and the prevalence of potential therapeutically accessible alterations, future studies will have to incorporate multi-site sampling and ideally single-cell DNA sequencing in order to build on these pivotal studies.

Regarding the critical prognosis of BPDCN, a prompt and reliable diagnosis of this rare entity is essential to assure accurate intensive induction or targeted therapy followed by consolidation with hematopoietic stem cell transplantation. Notably, patients undergoing front-line SCT achieved significantly better overall survival. So far, several studies have shown the superiority of allogeneic SCT over autologous SCT as consolidating therapy although all of them analyzed only small numbers of patients. In the study of Labiri et al., induction therapy of 61 and 16 patients was followed by allogeneic and autologous SCT, respectively. Patients who underwent allogeneic SCT did not reach median OS whereas OS was 65 months for patients undergoing autologous SCT; however, the sample sizes were highly dysbalanced and a relevant selection bias was evident [7]. The time point of allogeneic SCT is also important. Kharfan-Dabaja et al. [63] reported a pooled OS of 67% for patients undergoing allogeneic SCT at first complete remission (CR1) compared to 7% for patients who underwent allogeneic SCT beyond CR1 in a meta-analysis with 128 patients.

The overall prognosis of BPDCN patients remains unsatisfying, as only 35% of all patients were alive after a median follow-up of 10 months in our study. The anti-CD123 conjugated cytotoxic agent tagraxofusp represents a newly available option for induction therapy in BPDCN, especially for patients ineligible for an intensive chemotherapy regimen, and it was applied in 9% of our patients (most patients in our cohort were diagnosed before the compound’s approval). In stem cell transplantation (SCT) eligible patients, tagraxofusp can be applied as a bridging therapy to SCT, preferably to allogeneic SCT acknowledging the highest long-term survivorship of the latter in BPDCN. In those patients who are non-SCT eligible, tagraxofusp should be applied as an induction/re-induction and maintenance therapy [64]. After the introduction of tagraxofusp, rapid and correct diagnostics of BPDCN have become more evident than ever. Following the overexpression of BCL-2 in BPDCN and the role of epigenetics in its pathogenesis [65], the combination of BCL-2 inhibitor venetoclax and hypomethylating agent 5-azacytidine shows promising results in patients who fail tagraxofusp [18]. The limitations of our study are its retrospective character harboring the potential of fragmentary data, and patients were diagnosed in 5 different centers (albeit all with a high expertise level of hematopathology and specialized diagnostic procedures indispensable to diagnose BPDCN). At the same time, the strength of our study was a real-life scenario depicting diagnostic and clinical routing settings.

Given the low frequency of BPDCN and the fact that it commonly mimics other hematological neoplasms, the diagnosis of BPDCN still presents a relevant challenge. Multidisciplinary diagnostic boards and exchanges among hemato-oncologists, laboratory staff, pathologists, and dermatologists play an essential role in the correct and timely diagnosis of BPDCN. Immunophenotyping should include CD4, CD45, CD56, CD123, HLA-DR, and TCL-1 if BPDCN is suspected. Finally, education workshops, multicenter case collections, and rapidly available reference pathology may be crucial to further improve the diagnostic approaches in patients with suspected BPDCN.

## Data Availability

The data presented in this study are available on request from the corresponding author.

## References

[1] Swerdlow SH HN, Facchetti F et al (2008) Blastic plasmacytoid dendritic cell neoplasm. In: WHO classification of tumors of haematopoietic and lymphoid tissues. Lyon: IARC Press, pp 145–7

[2] NCCN clinical guidelines in oncology: myelodysplastic syndromes [Internet]. Available from: https://www.nccn.org/professionals/physician_gls/pdf/mds.pdf. Accessed 30 Sept 2023

[3] Fernandes F, Barreira R, Cortez J, Silveira M, Bain BJ (2018). The distinctive cytology and disease evolution of blastic plasmacytoid dendritic cell neoplasm. Am J Hematol.

[4] Arber DA, Orazi A, Hasserjian R, Thiele J, Borowitz MJ, Le Beau MM (2016). The 2016 revision to the World Health Organization classification of myeloid neoplasms and acute leukemia. Blood.

[5] Pagano L, Valentini CG, Pulsoni A, Fisogni S, Carluccio P, Mannelli F (2013). Blastic plasmacytoid dendritic cell neoplasm with leukemic presentation: an Italian multicenter study. Haematologica.

[6] Pagano L, Valentini CG, Grammatico S, Pulsoni A (2016). Blastic plasmacytoid dendritic cell neoplasm: diagnostic criteria and therapeutical approaches. Br J Haematol.

[7] Laribi K, Baugier de Materre A, Sobh M, Cerroni L, Valentini CG, Aoki T (2020). Blastic plasmacytoid dendritic cell neoplasms: results of an international survey on 398 adult patients. Blood Adv.

[8] Julia F, Petrella T, Beylot-Barry M, Bagot M, Lipsker D, Machet L (2013). Blastic plasmacytoid dendritic cell neoplasm: clinical features in 90 patients. Br J Dermatol.

[9] Martín-Martín L, Almeida J, Pomares H, González-Barca E, Bravo P, Giménez T (2016). Blastic plasmacytoid dendritic cell neoplasm frequently shows occult central nervous system involvement at diagnosis and benefits from intrathecal therapy. Oncotarget.

[10] Feng Z, Zhou J, Bentley G (2014). Blastic plasmacytoid dendritic cell neoplasm: report of a case presenting with lung and central nervous system involvement and review of the literature. J Louisiana State Med Soc.

[11] Aoki T, Suzuki R, Kuwatsuka Y, Kako S, Fujimoto K, Taguchi J (2015). Long-term survival following autologous and allogeneic stem cell transplantation for blastic plasmacytoid dendritic cell neoplasm. Blood.

[12] Martín-Martín L, López A, Vidriales B, Caballero MD, Rodrigues AS, Ferreira SI (2015). Classification and clinical behavior of blastic plasmacytoid dendritic cell neoplasms according to their maturation-associated immunophenotypic profile. Oncotarget.

[13] Bashir Q, Milton DR, Popat UR (2022). Allogeneic hematopoietic cell transplantation for patients with blastic plasmacytoid dendritic cell neoplasm (BPDCN). Bone Marrow Transplant.

[14] Roos-Weil D, Dietrich S, Boumendil A, Polge E, Bron D, Carreras E (2013). Stem cell transplantation can provide durable disease control in blastic plasmacytoid dendritic cell neoplasm: a retrospective study from the European Group for blood and marrow transplantation. Blood.

[15] Kharfan-Dabaja MA, Al Malki MM, Deotare U (2017). Haematopoietic cell transplantation for blastic plasmacytoid dendritic cell neoplasm: a North American multicentre collaborative study. Br J Haematol.

[16] Wilson NR, Konopleva M, Khoury JD, Pemmaraju N (2021). Novel therapeutic approaches in blastic plasmacytoid dendritic cell neoplasm (BPDCN): era of targeted therapy. Clin Lymphoma Myeloma Leuk.

[17] Samhouri Y, Ursu S, Dutton N, Tanvi V, Fazal S (2020). Tagraxofusp followed by combined azacitidine and venetoclax in blastic plasmacytoid dendritic cell neoplasm: a case report and literature review. J Oncol Pharm Pract.

[18] Tzankov A, Hebeda K, Kremer M, Leguit R, Orazi A, van der Walt J (2017). Plasmacytoid dendritic cell proliferations and neoplasms involving the bone marrow: summary of the workshop cases submitted to the 18th Meeting of the European Association for Haematopathology (EAHP) organized by the European Bone Marrow Working Group, Basel 2016. Ann Hematol.

[19] Garnache-Ottou F, Chaperot L, Biichle S, Ferrand C, Remy-Martin JP, Deconinck E (2005). Expression of the myeloid-associated marker CD33 is not an exclusive factor for leukemic plasmacytoid dendritic cells. Blood.

[20] Garnache-Ottou F, Feuillard J, Ferrand C, Biichle S, Trimoreau F, Seilles E (2009). Extended diagnostic criteria for plasmacytoid dendritic cell leukaemia. Br J Haematol.

[21] Kawai K (2005). CD56-negative blastic natural killer-cell lymphoma (agranular CD4(+)/CD56(+) haematodermic neoplasm)?. Br J Dermatol.

[22] Guolo F, Minetto P, Clavio M, Marcolin R, Miglino M, Passannante M (2020). Prognostic relevance of a blastic plasmacytoid dendritic cell neoplasm-like immunophenotype in cytogenetically normal acute myeloid leukemia patients. Leuk Lymphoma.

[23] Khanlari M, Yin CC, Takahashi K, Lachowiez C, Tang G, Loghavi S (2022). Bone marrow clonal hematopoiesis is highly prevalent in blastic plasmacytoid dendritic cell neoplasm and frequently sharing a clonal origin in elderly patients. Leukemia.

[24] Hamadeh F, Awadallah A, Meyerson HJ, Beck RC (2020). Flow cytometry identifies a spectrum of maturation in myeloid neoplasms having plasmacytoid dendritic cell differentiation. Cytometry B Clin Cytom.

[25] Garnache-Ottou F, Vidal C, Biichlé S, Renosi F, Poret E, Pagadoy M (2019). How should we diagnose and treat blastic plasmacytoid dendritic cell neoplasm patients?. Blood Adv.

[26] Menezes J, Acquadro F, Wiseman M, Gómez-López G, Salgado RN, Talavera-Casañas JG (2014). Exome sequencing reveals novel and recurrent mutations with clinical impact in blastic plasmacytoid dendritic cell neoplasm. Leukemia.

[27] Stenzinger A, Endris V, Pfarr N, Andrulis M, Jöhrens K, Klauschen F (2014). Targeted ultra-deep sequencing reveals recurrent and mutually exclusive mutations of cancer genes in blastic plasmacytoid dendritic cell neoplasm. Oncotarget.

[28] Sapienza MR, Pileri S (2020). Molecular features of blastic plasmacytoid dendritic cell neoplasm: DNA mutations and epigenetics. Hematol Oncol Clin North Am.

[29] Yin CC, Pemmaraju N, You MJ, Li S, Xu J, Wang W (2021). Integrated clinical genotype-phenotype characteristics of blastic plasmacytoid dendritic cell neoplasm. Cancers (Basel).

[30] (EMA) EMA (2020) Approval of the marketing authorisation for Elzonris (tagraxofusp). https://www.emaeuropaeu/en/documents/smop-initial/questions-answers-approval-marketing-authorisation-elzonris-tagraxofusp_enpdf. Accessed 30 Sept 2023

[31] Bôle-Richard E, Pemmaraju N, Caël B, Daguindau E, Lane AA (2022). CD123 and more: how to target the cell surface of blastic plasmacytoid dendritic cell neoplasm. Cancers.

[32] El Khawanky N, Hughes A, Yu W, Myburgh R, Matschulla T, Taromi S (2021). Demethylating therapy increases anti-CD123 CAR T cell cytotoxicity against acute myeloid leukemia. Nat Commun.

[33] Hammond D, Pemmaraju N (2020). Tagraxofusp for blastic plasmacytoid dendritic cell neoplasm. Hematol Oncol Clin North Am.

[34] Haase D, Germing U, Schanz J, Pfeilstocker M, Nosslinger T, Hildebrandt B (2007). New insights into the prognostic impact of the karyotype in MDS and correlation with subtypes: evidence from a core dataset of 2124 patients. Blood.

[35] Haase D, Feuring-Buske M, Könemann S, Fonatsch C, Troff C, Verbeek W (1995). Evidence for malignant transformation in acute myeloid leukemia at the level of early hematopoietic stem cells by cytogenetic analysis of CD34+ subpopulations. Blood.

[36] Braulke F, Jung K, Schanz J, Götze K, Müller-Thomas C, Platzbecker U (2013). Molecular cytogenetic monitoring from CD34+ peripheral blood cells in myelodysplastic syndromes: first results from a prospective multicenter German diagnostic study. Leuk Res.

[37] Pfeilstöcker M, Reisner R, Nösslinger T, Grüner H, Nowotny H, Tüchler H (1999). Cross-validation of prognostic scores in myelodysplastic syndromes on 386 patients from a single institution confirms importance of cytogenetics. Br J Haematol.

[38] McGowan-Jordan J, Simons A, Schmid M (2016). ISCN: an international system for human cytogenomic nomenclature (2016).

[39] Simons A, Shaffer LG, Hastings RJ (2013). Cytogenetic nomenclature: changes in the ISCN 2013 compared to the 2009 edition. Cytogenet Genome Res.

[40] Martin R, Acha P, Ganster C, Palomo L, Dierks S, Fuster-Tormo F (2018). Targeted deep sequencing of CD34+ cells from peripheral blood can reproduce bone marrow molecular profile in myelodysplastic syndromes. Am J Hematol.

[41] Künstner A, Schwarting J, Witte HM, Bernard V, Stölting S, Kusch K (2022). Integrative molecular profiling identifies two molecularly and clinically distinct subtypes of blastic plasmacytoid dendritic cell neoplasm. Blood Cancer J.

[42] Cuglievan B, Connors J, He J, Khazal S, Yedururi S, Dai J (2023). Blastic plasmacytoid dendritic cell neoplasm: a comprehensive review in pediatrics, adolescents, and young adults (AYA) and an update of novel therapies. Leukemia.

[43] Wang W, Khoury JD, Miranda RN, Jorgensen JL, Xu J, Loghavi S (2021). Immunophenotypic characterization of reactive and neoplastic plasmacytoid dendritic cells permits establishment of a 10-color flow cytometric panel for initial workup and residual disease evaluation of blastic plasmacytoid dendritic cell neoplasm. Haematologica.

[44] Renosi F, Roggy A, Giguelay A, Soret L, Viailly P-J, Cheok M (2021). Transcriptomic and genomic heterogeneity in blastic plasmacytoid dendritic cell neoplasms: from ontogeny to oncogenesis. Blood Adv.

[45] Rodrigues PF, Alberti-Servera L, Eremin A, Grajales-Reyes GE, Ivanek R, Tussiwand R (2018). Distinct progenitor lineages contribute to the heterogeneity of plasmacytoid dendritic cells. Nat Immunol.

[46] Narducci MG, Pescarmona E, Lazzeri C, Signoretti S, Lavinia AM, Remotti D (2000). Regulation of TCL1 expression in B- and T-Cell lymphomas and reactive lymphoid tissues1. Can Res.

[47] Ceribelli M, Hou Zhiying E, Kelly Priscilla N, Da Huang W, Wright G, Ganapathi K (2016). A druggable TCF4- and BRD4-dependent transcriptional network sustains malignancy in blastic plasmacytoid dendritic cell neoplasm. Cancer Cell.

[48] Boiocchi L, Lonardi S, Vermi W, Fisogni S, Facchetti F (2013). BDCA-2 (CD303): a highly specific marker for normal and neoplastic plasmacytoid dendritic cells. Blood.

[49] Pemmaraju N, Wilson NR, Khoury JD, Jain N, Daver N, Pierce S (2021). Central nervous system involvement in blastic plasmacytoid dendritic cell neoplasm. Blood.

[50] Martín-Martín L, Almeida J, Pomares H (2016). Blastic plasmacytoid dendritic cell neoplasm frequently shows occult central nervous system involvement at diagnosis and benefits from intrathecal therapy. Oncotarget.

[51] Taylor J, Haddadin M, Upadhyay VA, Grussie E, Mehta-Shah N, Brunner AM (2019). Multicenter analysis of outcomes in blastic plasmacytoid dendritic cell neoplasm offers a pretargeted therapy benchmark. Blood.

[52] Herling M, Jones D (2007). CD4+/CD56+ hematodermic tumor: the features of an evolving entity and its relationship to dendritic cells. Am J Clin Pathol.

[53] Tsagarakis NJ, Paterakis G (2020). Dendritic cell leukemia: a review. Curr Oncol Rep.

[54] Maria Rosaria S, Francesco A, Federica M, Stefania O, Fabio F, Maryam E (2019). Blastic plasmacytoid dendritic cell neoplasm: genomics mark epigenetic dysregulation as a primary therapeutic target. Haematologica.

[55] Witte HM, Künstner A, Schwarting J, Bernard V, Stölting S, von Bubnoff N (2022). Genome-wide DNA methylation profiling in blastic plasmacytoid dendritic cell neoplasm. Blood.

[56] Xiao W, Chan A, Waarts MR, Mishra T, Liu Y, Cai SF (2021). Plasmacytoid dendritic cell expansion defines a distinct subset of RUNX1-mutated acute myeloid leukemia. Blood.

[57] Xiao W, Goldberg AD, Famulare C, Baik J, Gao Q, Tallman MS (2018). Acute myeloid leukemia with plasmacytoid dendritic cell differentiation: predominantly secondary AML, enriched for RUNX1 mutations, frequent cross-lineage antigen expression and poor prognosis. Blood.

[58] Togami K, Chung SS, Madan V, Booth CAG, Kenyon CM, Cabal-Hierro L (2022). Sex-biased ZRSR2 mutations in myeloid malignancies impair plasmacytoid dendritic cell activation and apoptosis. Cancer Discov.

[59] Griffin GK, Booth CAG, Togami K, Chung SS, Ssozi D, Verga JA (2023). Ultraviolet radiation shapes dendritic cell leukaemia transformation in the skin. Nature.

[60] Wang L, Yang M, Zhang X, Yang C, Huang X, Wang Z (2017). ARID1A mutation in blastic plasmacytoid dendritic cell neoplasm. Haematologica.

[61] Kharfan-Dabaja MA, Reljic T, Murthy HS, Ayala E, Kumar A (2018). Allogeneic hematopoietic cell transplantation is an effective treatment for blastic plasmacytoid dendritic cell neoplasm in first complete remission: systematic review and meta-analysis. Clin Lymphoma Myeloma Leuk.

[62] Pagano L, Zinzani PL, Pileri S, Quaglino P, Cuglievan B, Berti E (2023). Unmet clinical needs and management recommendations for blastic plasmacytoid dendritic cell neoplasm: a consensus-based position paper from an ad hoc international expert panel. Hemasphere.

[63] Sapienza MR, Fuligni F, Agostinelli C, Tripodo C, Righi S, Laginestra MA (2014). Molecular profiling of blastic plasmacytoid dendritic cell neoplasm reveals a unique pattern and suggests selective sensitivity to NF-kB pathway inhibition. Leukemia.

[64] Montero J, Stephansky J, Cai T, Griffin GK, Cabal-Hierro L, Togami K (2017). Blastic plasmacytoid dendritic cell neoplasm is dependent on BCL2 and sensitive to venetoclax. Cancer Discov.

[65] Gangat N, Konopleva M, Patnaik MM, Jabbour E, DiNardo C, Al-Kali A (2022). Venetoclax and hypomethylating agents in older/unfit patients with blastic plasmacytoid dendritic cell neoplasm. Am J Hematol.

